# Retrospective Cohort Study of the Prevalence of Lumbosacral Transitional Vertebra in a Wide and Well-Represented Population

**DOI:** 10.1155/2013/461425

**Published:** 2013-06-24

**Authors:** Demet Uçar, Bekir Yavuz Uçar, Yahya Coşar, Kurtuluş Emrem, Gürkan Gümüşsuyu, Serhat Mutlu, Burcu Mutlu, Mehmet Akif Çaçan, Yılmaz Mertsoy, Hatice Gümüş

**Affiliations:** ^1^Department of Physical Medicine and Rehabilitation, Dicle University Medical Faculty, Diyarbakir, Turkey; ^2^Department of Orthopaedics and Traumatology, Dicle University Medical Faculty, Diyarbakir, Turkey; ^3^Department of Orthopaedics and Traumatology, Beyşehir Government Hospital, Konya, Turkey; ^4^Department of Orthopaedics and Traumatology, Güngören Hospital, İstanbul, Turkey; ^5^Department of Orthopaedics and Traumatology, Terme Government Hospital, Samsun, Turkey; ^6^Department of Orthopaedics and Traumatology, Nisa Hospital, İstanbul, Turkey; ^7^Department of Physical Medicine and Rehabilitation, Nisa Hospital, İstanbul, Turkey; ^8^Department of Radiology, Dicle University Medical Faculty, Diyarbakir, Turkey

## Abstract

*Purpose*. The aim of this study is to determine the prevalence of lumbosacral transitional vertebra (LSTV) in a well-represented general population. *Methods*. For a retrospective cohort study, abdominal radiographs of adult subjects were queried with clear visibility of the vertebral body articulation of the last rib, all lumbar transverse processes, and complete sacral wings. Exclusion criteria included any radiologic evidence of previous lumbosacral surgery that would block our view. A total of 6200 abdominal films were reviewed, and 3607 were identified as being suitable for the measurement of the desired parameters. *Results*. A total of 3607 subjects were identified as eligible for the study, and 683 (18.9%) were classified as positive for a lumbosacral transitional vertebra. The prevalence of sacralization and lumbarization was found as 17.2% and 1.7%, respectively. The average age at the time of the study was 39.5 ± 15.2 years (18–86 years). *Conclusions*. As a result of different opinions, LSTV retains its controversial status. Our prevalence study of the general population will provide assistance for resolution of the controversy. Prevalence studies of the general population with a wide participation will shed light on comparative studies.

## 1. Introduction

Lumbosacral transitional vertebra (LSTV) is a congenital vertebral anomaly of the L5-S1 junction in the spine [1]. LSTV occurs because of sacralization in which one or both of the transverse processes of the fifth lumbar vertebra exhibits fusion with the first sacral segment or as a result of lumbarization in which the first sacral segment exhibits an abnormal transverse process similar to that of the lumbar vertebra. This modification may contribute to incorrect determination of a vertebral segment, which can lead to surgery at the wrong level and unresolved symptoms [1].

LSTV is evaluated with lumbosacral anteroposterior radiographs, and abdominal radiographs may also show this anomaly. Abdominal radiographs, as radiographic examinations for abdominal indications, have many clinical uses and are most often indicated for patients who exhibit signs of intestinal obstruction or visceral perforation. In this study, a different purpose was pursued. We used abdominal radiographs which were with clear visibility of the last rib's vertebral body articulation, all lumbar transverse processes, and the complete sacral wing to provide radiographic images of the lumbar spine. For a study evaluating LSTV cases, the use of these radiographs was desirable as they are likely to be representative of the general population. The coexistence of low back pain and LSTV can create bias for this prevalence study. In order to eliminate low back pain population, we did not prefer lumbosacral radiographs.

Several studies have been reported about the LSTV in a back pain population [2–6]; however, the prevalence studies with a wide participation of members of the general populations are lacking. Therefore, our study aims to establish the prevalence rates for LSTV in the wide and well-represented population.

## 2. Methods

A retrospective cohort study was conducted by reviewing the abdominal radiographs of 6200 patients over a one-year period after the institution review board has been approved. These were identified from four cities (İstanbul, Diyarbakır, Konya, and Samsun) in different geographic regions of our country (Turkey). Inclusion criteria were subject's age older than 18 years at time of radiographs and abdominal radiographs available with clear visibility of the last rib's vertebral body articulation, all lumbar transverse processes, and the complete sacral wing. Exclusion criteria consisted of any radiologic evidence of previous lumbosacral surgery that would block our view. A total of 6200 abdominal radiographs from the past year (2011) were reviewed, and 3607 were identified as being suitable for the measurement of the desired parameters as we described the clear visibility in this retrospective cohort study.

Data collection consisted of the subject's age at the time of imaging, gender, bilateral height measurement of the lowest lumbar transverse processes, and the classification of the sacralization. Three spine specialists and one radiologist performed all of the measurements and classified the cases, using a systemized approach according to the Castellvi [7] radiographic classification system (Table 1) to decrease variability; in addition, the reviewers consulted among themselves. They read all the radiographs and evaluated the reproducibility of the descriptions. They determined the interobserver reliability. Digital films were downloaded to an imaging processing program for standardization of the measurements.

The prevalence of LSTV, sacralization, and lumbarization was reported separately. The anomalies were compared according to gender.

Statistically significant differences were evaluated using the chi-square test for categorical variables (gender), and significance was set at *P* < 0.05.

## 3. Results

**Figure 1:**
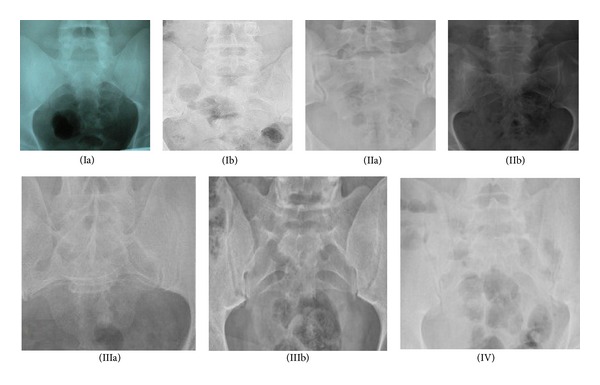
Types of the Castellvi radiographic classification system. (Ia) Case number 1746. (Ib) Case number 312. (IIa) Case number 1021. (IIb) Case number 1290. (IIIa) Case number 1075. (IIIb) Case number 1330. (IV) Case number 1988.

A total of 1843 female and 1764 male subjects were identified as eligible for the study; the average age was 39.5 ± 15.2 years (18–86 years). Average ages of the subjects according to the classification types were shown in Table 2. Of these subjects, 683 were classified as positive for LSTV (Figure 1), with a gender distribution of 314 (46%) women and 369 (54%) men. The prevalence was found as 18.9% (Table 3).

A total of 623 subjects were classified as positive for sacralization, with a gender distribution of 276 (44.5%) women and 344 (55.5%) men, for a prevalence of 17.2% (Table 4). The most common anatomical variant was Castellvi type Ia (5.5%). A total of 63 lumbarizations were classified, for a prevalence of 1.7%.

Statistically significant differences were found between the two sex groups in subjects with LSTV (*P* = 0.002) and sacralization (*P* < 0.001). Prevalence of lumbarization was higher in women but the difference was not statistically significant (*P* = 0.088). Higher incidences of type Ia and type Ib were found in men and these differences were statistically significant (*P* = 0.016, *P* < 0.001) when compared with the women (Table 4).

## 4. Discussion

Lumbosacral transitional vertebral frequency of the general population has been given a wide range of percentages ranging from 4% to 36% [1, 8–11]. Most studies have evaluated the relationship between low back pain and LSTV [4, 5, 7, 12–15]. Few further studies have been published regarding racial differences [11, 16, 17]. The wide range observed may be explained by differences in diagnostic criteria, imaging techniques, and confounding factors between the investigated population samples. Hsieh et al. [16] found a prevalence of 4% in a population consisting primarily of Chinese patients when using anteroposterior (AP) plain radiographs for diagnosis. However, they excluded Castellvi type I. Erken et al. [17] also used AP plain radiographs for diagnosis but did not exclude subtypes of LSTV. They found a prevalence of 35.9% in the Turkish population sample. Apazidis et al. [11] examined 211 subjects (107 men and 104 women) and found that 75 were classified as positive for transitional lumbosacral vertebra, with a gender distribution of 40 (19%) men and 35 (16.6%) women. They reported the prevalence at 35.6% in the American general population. Hsieh and Erken both used lumbosacral radiographs for their studies. The study of Apazidis et al. used kidney-urinary bladder radiographs. In the present study, we used abdominal radiographs instead of lumbosacral graphs. The coexistence of low back pain and LSTV can create bias for this prevalence study. In order to eliminate low back pain population, we did not prefer lumbosacral graphs. The prevalence of LSTV in the general population was found as 18.7% in our study.

Igbinedion and Akhigbe [18] reported a higher incidence of sacralization in men and a higher incidence of lumbarization in women, but no statistical correlation was established between transitional vertebra and gender. Magora and Shwartz [19] found an overall incidence of 0.65% lumbarization and 20.8% sacralization, and the incidence was markedly lower in women. They reported no direct relation between sacralization and age, sex, or ethnic community. Olanrewaju [20] showed that sacralization is predominant in males. Despite the higher number of females present in the sample population, more males were seen to have LSTV and also presented with sacralization. He recorded a relationship between LSTV and gender. In our study, the occurrence of type Ia and Ib was statistically higher in men. LSTV has been reported to occur with a higher statistical prevalence in male subjects. The significant sex effect reflects body size gender dimorphism in humans. This reveals the uniqueness of each case and may reflect the numerous and complicated genetic and developmental factors involved. The width of the last lumbar transverse process is likely related to the sacral characteristics. Having a statistically significantly higher prevalence of type 1a and 1b in male subjects can be explained by gender dimorphism.

The LSTV frequency in the low back pain (LBP) population ranges from 6% to 37% [4, 5, 7, 12–15]. The relationship between LBP and LSTV is not clear. Numerous studies have found no significant correlation between transitional vertebrae and low back pain [3, 5, 6, 9], while others have found such a correlation [7, 8, 12, 15, 19]. Wigh and Anthony Jr. [12] and Castellvi et al. [7] advocated a relationship between LSTV and LBP. Frymoyer et al. [3] determined similar rates of radiological abnormalities in three groups of patients—no LBP, moderate, and severe. Similarly, Otani et al. [9] reported the incidence of transitional vertebra to be 13% in patients with LBP and 11% in their control group. Our study presented a prevalence of LSTV in a well-represented general population as a contribution to help resolve this controversy.

## 5. Conclusion

As a result of different research opinions, LSTV retains its controversial status. In this study, we wanted to find the prevalence of this pathology and the relationship between the genders. Our retrospective cohort study of the prevalence of a wide and well-represented population was found compatible with the literatures in which a wide range of frequency in the previous studies have been reported. The limitation of our study was literally not reflecting the general population. Prevalence studies of the population with wide participation such as the present case will help to shed light on future comparative studies.

## Figures and Tables

**Table 1:** Castellvi radiographic classification system of sacralization [7].

Type Ia	A unilateral TP height greater than or equal to 19 mm
Type Ib	Both processes heights greater than or equal to 19 mm
Type IIa	Presence of unilateral articulation between the TP and the sacrum
Type IIb	Presence of bilateral articulation between the TP and the sacrum
Type IIIa	Unilateral fusion of the TP and the sacrum
Type IIIb	Bilateral fusion of the TP and the sacrum
Type IV	Unilateral type II transition (articulation) with a type III (fusion) on the contralateral side

TP: lowest lumbar transverse process.

**Table 2:** Average ages of the subjects according to the classification types.

	*n*	Mean age (years)
Type Ia	200	43 ± 15.8
Type Ib	135	40.6 ± 13.9
Type IIa	75	37.5 ± 12.5
Type IIb	50	36 ± 13.2
Type IIIa	36	38.9 ± 12,8
Type IIIb	83	37.4 ± 15.5
Type IV	41	38.1 ± 17

**Table 3:** Numbers of cases and prevalences of the anomalies.

	*n*	Mean age (years)	Prevalence of LSTV (%)	Prevalence of sacralization (%)	Prevalence of lumbarization (%)
Women	1843	39.7 ± 15.3	17	15	2.1
Men	1764	39.4 ± 15.1	20.9	19.5	1.4
Total	3607	39.5 ± 15.2	18.9	17.2	1.7

LSTV: lumbosacral transitional vertebra.

**Table 4:** Comparison of the classification according to gender.

	Women (*n*)	Men (*n*)	*P* value*
Type Ia	87	113	0.016
Type Ib	47	88	<0.001**
Type IIa	34	41	0.186
Type IIb	22	28	0.193
Type IIIa	21	15	0.241
Type IIIb	41	42	0.42
Type IV	24	17	0.212
Lumbarization	38	25	0.088
Sacralization	276	344	<0.001**
LSTV	314	369	0.002**

LSTV: lumbosacral transitional vertebra.

*Chi-square test.

**Statistically significant.
